# Melatonin pre-treatment mitigates SHSY-5Y cells against oxaliplatin induced mitochondrial stress and apoptotic cell death

**DOI:** 10.1371/journal.pone.0180953

**Published:** 2017-07-21

**Authors:** Mohammad Waseem, Upasana Sahu, Mohd. Salman, Arnab Choudhury, Sudeshna Kar, Heena Tabassum, Suhel Parvez

**Affiliations:** 1 Department of Medical Elementology and Toxicology, Jamia Hamdard (Hamdard University), New Delhi, India; 2 Jamia Hamdard-Institute of Molecular Medicine, Jamia Hamdard (Hamdard University), New Delhi, India; 3 Department of Biochemistry, Jamia Hamdard (Hamdard University), New Delhi, India; University of Manitoba, CANADA

## Abstract

Oxaliplatin (Oxa) treatment to SH-SY5Y human neuroblastoma cells has been shown by previous studies to induce oxidative stress, which in turn modulates intracellular signaling cascades resulting in cell death. While this phenomenon of Oxa-induced neurotoxicity is known, the underlying mechanisms involved in this cell death cascade must be clarified. Moreover, there is still little known regarding the roles of neuronal mitochondria and cytosolic compartments in mediating Oxa-induced neurotoxicity. With a better grasp of the mechanisms driving neurotoxicity in Oxa-treated SH-SY5Y cells, we can then identify certain pathways to target in protecting against neurotoxic cell damage. Therefore, the purpose of this study was to determine whether one such agent, melatonin (Mel), could confer protection against Oxa-induced neurotoxicity in SH-SY5Y cells. Results from the present study found Oxa to significantly reduce SH-SY5Y cell viability in a dose-dependent manner. Alternatively, we found Mel pre-treatment to SH-SY5Y cells to attenuate Oxa-induced toxicity, resulting in a markedly increased cell viability. Mel exerted its protective effects by regulating reactive oxygen species (ROS) production and reducing superoxide radicals inside Oxa-exposed. In addition, we observed pre-treatment with Mel to rescue Oxa-treated cells by protecting mitochondria. As Oxa-treatment alone decreases mitochondrial membrane potential (Δψm), resulting in an altered Bcl-2/Bax ratio and release of sequestered cytochrome c, so Mel was shown to inhibit these pathways. Mel was also found to inhibit proteolytic activation of caspase 3, inactivation of Poly (ADP Ribose) polymerase, and DNA damage, thereby allowing SH-SY5Y cells to resist apoptotic cell death. Collectively, our results suggest a role for melatonin in reducing Oxa induced neurotoxicity. Further studies exploring melatonin’s protective effects may prove successful in eliciting pathways to further alter the neurotoxic pathways of platinum compounds in cancer treatment.

## Introduction

Neurotoxicity is one major side effect seen in platinum-based chemotherapy that ultimately counteracts the administration of an effective dose and regularly prompts patients to withdraw treatment. Importantly, platinum derivatives are among the most frequently utilized anticancer agents. One such drug, Oxaliplatin (Oxa), has been shown to cause higher cytotoxicity and produce less DNA adducts compared to CP (cisplatin) at equimolar concentrations [1]. Oxa, a third-era platinum analogue, is typically used in clinical practice to treat various cancers of the gastrointestinal tract. However, the significant neurotoxicity induced by this drug at potential measurements may also constrain the reaction of treatment [1–3].

Neurotoxicity in the setting of acute exposures may emerge following the first dose. In addition, symptoms such as cold-provoked paresthesias and withdrawal cramps may also appear within 1 week of the last administered dose. It is also possible for chronic, painful symptoms to develop and reappear sporadically in the months following treatment discontinuation, namely as a result of lingering Oxa-accumulation in the body. [4, 5]. The possible causes of Oxa induced neurotoxicity (both acute and chronic) are still not completely understood.

*In vitro* studies have displayed significant alterations in cellular structure and function of Oxa-treated neuronal cells [6, 7]. Neurotoxicity has been investigated *in vitro* using various cellular and molecular approaches. Several reports have been published that also utilize tumor cell lines such as SH-SY5Y human neuroblastoma cells. These cellular models have proven to be applicable for studying molecular mechanisms involved in neurotoxicity induced by Oxa [1, 8]. It seems the alterations in cellular structure and function may play an important role in elucidating various aspects of Oxa neurotoxicity. In response to anti-cancer agents such as Oxa, cells have been reported to respond with increased generation of ROS, ultimately leading to the activation of apoptotic pathways [8, 9]. Further, this ROS production during chemotherapy treatment may also be responsible for serious toxic events including neurotoxicity [9]. Oxidative stress generated in this context causes cytotoxicity to neurons by inducing demyelination, mitochondrial dysfunction, microtubular damage, and apoptosis in neurons [10, 11].

A range of therapeutic agents derived from nutraceuticals have revealed neuroprotective action in both *in vitro* and *in vivo* models of neuronal cell death or neurodegeneration [12, 13]. It has been reported that Mel, a pineal hormone, as well as its metabolites, display important antioxidant properties in the nervous system [13, 14]. Mel has been previously recognized for its protective effects, which include properties related to radical scavenging and antioxidant potential [10]. As a consequence of its lipophilic nature, Mel is able to cross all cell membranes, allowing it to reach and accumulate in sub-cellular compartments including nuclei and mitochondria [14]. Several investigators have demonstrated Mel to promptly rescue the mitochondria from oxidative stress induced dysfunction, and also prevent the resultant apoptotic events and death in neuronal cell lines. A prior mechanistic study at mitochondrial levels found Mel to inhibit the action of oxidative stress-induced permeability transition pore (PTP) opening in astrocytes, which in the absence of protective factors would instigate mitochondrial and nuclear DNA damage [15]. Recently, Mel has also been shown to modulate PTP and 5-hydroxydecanoate-induced K_ATP_ channel inhibition in isolated brain mitochondria [16]. However, there is still little data in literature regarding the neuroprotective effects of Mel against Oxa induced cell injury/ cell death. Thus, this study was designed to investigate the therapeutic potential of Mel in regulating the oxaliplatin- induced neurotoxic pathways in SH-SY5Y cells.

## Material and methods

### Chemicals

T-75 flasks and culture dishes (60 × 15 mm) were purchased from Corning, Inc. (Corning, NY). Twenty-four-well and 96-well plates were purchased from Beckton Dickson (Franklin Lakes, NJ). Calcein, Disodium hydrogen phosphate, 2′, 7′-Dichlorofluorescin diacetate (H2-DCFDA) and dihydroethidium (DHE), Dimethyl sulfoxide (DMSO), Dulbecco's Modified Eagle's Medium (DMEM) ethanol, and Fetal bovine serum (FBS) were purchased from Invitrogen, USA and Sigma Chemicals, MO, USA and E. Merck, India respectively. Methanol, 3-(4, 5-dimethylthiazol-2-yl)-2, 5-diphenyl-terazolium bromide (MTT), Penicillin, Phophatase inhibitor cocktail, Protease inhibitor cocktail, and Ponceau were purchased from Sigma Chemicals, MO, USA, E. Merck, India and Invitrogen, USA respectively. Sodium dihydrogen phosphate, Streptomycin, and Tween 80 were purchased from E. Merck, India and Invitrogen, USA. Tetramethylrhodamine ethyl ester (TMRE) was purchased from Sigma Chemicals, MO, USA. Rabbit anti-cytochrome c antibody (Cell Signaling Technology, MA, USA), rabbit anti-caspase 3 antibody (Cell Signaling Technology, MA, USA), rabbit anti-cleaved caspase antibody (Cell Signaling Technology, MA, USA), rabbit anti- Poly(ADP Ribose) polymerase antibody (PARP, Cell Signaling Technology, MA, USA), rabbit anti- cleaved Poly(ADP Ribose) polymerase antibody (PARP, Cell Signaling Technology, MA, USA), rabbit anti-Bax antibody (Santa Cruz Biotechnology, Inc., USA), mouse anti-Bcl-2 antibody (Santa Cruz Biotechnology, Inc., USA) and rabbit anti-GAPDH antibody (Cell Signaling Technology, MA, USA). Oxaliplatin and Melatonin were obtained from Sigma Co. (St. Louis, MO, USA).

### Cell lines and culture condition

Human neuroblastoma cell line, SH-SY5Y was obtained from the National Centre for Cell Science (NCCS), Pune, India. Stock cultures of SH-SY5Y cells (passages 26 to 30) were maintained in T-75 flasks. SH-SY5Y cells were grown in DMEM medium boosted with 10% FBS (Gibco, Invitrogen, USA), 100 U/ ml penicillin and 100 mg/ml streptomycin (Gibco, Invitrogen, USA) in a humidified incubator at 37°C with 5% CO2. The culture media was replaced every two to three days.

### Experimental design

All experiments were carried out 24 h after SH-SY5Y cells were seeded in culture plates. Oxa was dissolved in water to produce a stock solution of 1 mM; Mel was dissolved in 50% ethanol in water to obtain a 10 mM stock solution. Stock solutions were then diluted appropriately in culture media at the time of treatment. To induce cell injury or cytotoxicity, cells were treated for 24 h with different concentrations of Oxa (10 μM, 50 μM and 100 μM), that were predicted to be equivocal to therapeutic levels used in humans [17]. The concentrations of Oxa were based on an *in vitro* study with a neuronal cell line [1]. Each experiment was repeated a minimum of three times. For evaluation of Oxa induced neuronal toxicity and the modulating effects of Mel, cells were divided in four groups. Group I served as control (cells treated with ethanol), group II cells were treated with 10 μM Mel alone, group III cells were treated with 10 μM, 50 μM and 100 μM Oxa, and group IV cells received Mel pretreatment followed by Oxa (10 μM Mel 1 h prior to the treatment of 10 μM, 50 μM and 100 μM Oxa). In some experiments, only one selected concentration of Oxa was used for testing the protective effect of Mel. The concentrations of Mel were selected in accordance with previously published reports [18].

### Cell viability and cytotoxicity assays

#### Cell viability

The effect of Mel and Oxa on SH-SY5Y cell line was determined by a MTT dye- uptake method described in previous published report [19]. Cell viability was measured based on the alteration of MTT to purple formazan contents by mitochondrial dehydrogenases. Concisely, 5 × 104 cells/well were seeded on 96-well tissue culture plates and allowed to adhere for 24 h in 5% CO2 incubator at 37 0C. Subsequently, the cells were then pre-incubated with 10 μM Mel for 1 h followed with or without Oxa (10 μM, 50 μM and 100 μM) for 24 h in their respective medium. The dose of Mel and Oxa were based on previously published reports [1, 18]. After this step, half of the media was replaced by DMEM with MTT (0.5 mg/ml) and kept at 37 0C for 1 h. Formazan crystals were then solubilized with MTT reagent (0.004 M HCl and 1 mg/ml NP-40 in isopropanol) and kept for 20 min at 37 0C. Suspension was then transferred to a 96-well tissue culture plate and OD was computed at 570 nm by using Gen 5.0 software provided together with plate reader (BioTek Power Wave XS2, Winooski, VT, USA). MTT solvent was employed as blank. Each experiment was performed in triplicate and repeated three times in cultured cells. The results were expressed as percentage of controls.

#### Cytotoxicity

Lactate dehydrogenase (LDH) was measured to check for cytotoxic cell damage in Mel and Oxa treated SH-SY5Y cells. Cytotoxicity was measured using a LDH assay kit (Cayman Chemical, Ann Arbor, MI, USA). LDH is a cytosolic soluble enzyme that is retained by viable cells with intact plasma membranes but released in the event of membrane damage. Therefore, it is a useful indicator of disruption in cell membrane integrity that resulting from necrosis or apoptosis [20]. This stable enzyme was measured colorimetrically based on the conversion of lactate to pyruvate in the presence of NAD+ coupled to the conversion of a tetrazolium salt into a pink formazan product [21]. Briefly, 5 × 104 cells/well were seeded on 96-well tissue culture plates and allowed to adhere for 24 h in 5% CO2 incubator at 37 0C. Cells were treated with 10 μM Mel for 1 h, followed by different doses of Oxa and incubated for 24 h. The plate was spun at 2000 rpm for 2 mins to settle down dead and floating cells and 100 μl of culture supernatant was transferred to respective wells of a fresh 96 well plate. LDH activity was measured according to the manufacturer’s protocol at 24 h post treatment with Oxa. Absorbance was measured at 490 nm with a microplate reader (BoiTek Power Wave XS2, Winooski, VT, USA) with a reference wavelength of 630 nm. The results were expressed as μU/ml of LDH activity in treated and untreated cells.

### Fluorescence live cell assay

The cell survival assay was performed by using Live Cell Assay Calcein AM kit (Invitrogen, Waltham, Massachusetts, USA). This assay is based on the simultaneous determination of live cells by Calcein AM. Briefly, 2 × 105 cells/well were seeded on a 24 well tissue culture plate and treated with Oxa and Mel in their respective medium. Cells were washed three times with PBS, and incubated with Calcein AM (5 μM) in the dark for 30 min at room temperature. After incubation, 5–6 random images were captured from each well using an inverted microscope (Nikon Ts Eclipse, Japan). Live cells were estimated using FITC (485 nm) for the assessment of calcein fluorescence, produced by the cleavage of the calcein-AM ester by ubiquitous intracellular esterases to highly green fluorescent calcein.

### Morphology of cells (Bright field imaging)

SH-SY5Y cells (2 × 105 cells/well) were seeded in 24 well tissue culture plates and further treated with Oxa and Mel in their respective medium. Cell images were captured from 6 random fields at 20x magnification using NIS elements software provided with Nikon Ts Eclipse inverted microscope.

### Fluorometric measurement of mitochondrial membrane potential (Δψm)

Imaging or fluorometric measurement of mitochondrial membrane potential in intact SH-SY5Y cells was achieved by using membrane potential dependent fluorescent TMRE (Sigma, USA) probe [22]. This cationic, lipophilic dye enters cells in the form of an ester. It is then hydrolyzed to produce tetramethylrhodamine, a product that accumulates within the mitochondria due to a high membrane potential. Cells were seeded at a density of 5 × 105 in two well chamber slide for 24 h in 5% CO2 incubator at 37 0C. Thereafter, cells were pre- treated with 10 μM Mel for 1 h and later with or without 50 μM Oxa for 24 h. Cells were then washed twice with PBS and further stained or incubated with TMRE (100 nM) for 15 min at 37 0C. The stained cells were visualized with excitation at 540 nm and emission at 590 nm by using NIS elements software provided with Nikon Ts Eclipse inverted microscope. Five to six images were captured at 20x magnification. Image scales were burned on to each picture using the same software. Maximum projections were generated from focus planes and images were displayed with equal pixel intensity. The TMRE fluorescence integrated density was analyzed with Image J software (1.50 version, NIH, USA).

### Fluorometric measurement of intracellular ROS accumulation

The oxidation-sensitive fluorescent dye 2′, 7′- dichlorodihydrofluorescein diacetate (DCFH-DA, Sigma, USA) was utilized to quantify the production of intracellular ROS, hydrogen peroxide, and hydroxyl radicals as previously described [22]. This non-fluorescent product was then converted by reactive species into DCF, which could be easily visualized by fluorescence at 530 nm when excited at 485 nm. Briefly, SH-SY5Y cells (5 × 105 cells/well) were seeded in 6-well tissue culture plates and allowed to adhere for 24 h in 5% CO2 incubator at 37 0C. Thereafter, cells were treated with Oxa and Mel in their medium and incubated or stained with the fluorescent DCFH-DA (10 μg/ml) probe at 37 0C for 15 min. The fluorescence signal or images were recorded using FITC (488 nm) with NIS elements software provided with Nikon Ts Eclipse inverted microscope. The fluorescence signal was measured as proportional to ROS production. The DCF fluorescence integrated density was analyzed with Image J software (1.50 version, NIH, USA). Five to six random images were captured by using 20x objective lens. Image scales were burned on to each picture using the same software. Maximum projections were generated from focus planes and images were displayed with equal pixel intensity.

### Fluorometric measurement of superoxide anion production

Superoxide anion production was detected using the dihydroethidium (DHE) probe. 5 × 105 cells/well were seeded on 6-well tissue culture plates and allowed to adhere for 24 h in a 5% CO2 incubator at 37 0C. Thereafter, cells were treated with Oxa and Mel in their respective medium. Further, cells were stained or incubated with DHE solution (2 μg/ml) for 20 min. DHE dye incorporated into the cells and subsequently oxidized to the fluorescent ethidium cation by superoxide anion, allowing the cation to bind nuclear DNA with an extensive enhancement of fluorescence at 530 nm when excited at 485 nm [23]. The fluorescence signal was detected as proportional to superoxide production. The DHE fluorescence integrated density was analyzed with Image J software (1.50 version, NIH, USA). The 3–4 images were captured using NIS elements software provided by the Nikon Ts Eclipse inverted microscope at 20x magnification. Maximum projections were generated from focus planes and images were displayed with equal pixel intensity. Results were expressed as percentage of DHE stained positive cells.

### Annexin V/ 7-AAD based apoptosis assay by flow cytometry

Flow cytometric analysis was performed using PE Annexin V Apoptosis Detection Kit I (BD Biosciences, USA) following manufacturer’s instruction. For apoptosis and necrosis assays, 5 × 10^5^ cells/well were seeded in 6-well plates and treated with Oxa and Mel in their respective medium. Cells were then briefly washed three times with chilled PBS and collected by centrifugation for 5 min at 800 *g* at 4°C. Thereafter, cells were re-suspended in binding buffer as per manufacture’s instruction. 100 μl of cell suspension (1 × 10^5^ cells) were incubated with Annexin V PE (5 μl) and 7-AAD (5 μl) for 15 min in dark condition. 400 μl of 1X binding buffer was added to each tube and samples were analyzed by flow cytometry using BD LSR II (Becton Dickinson, USA) and data analyses were done using BD FACSDiva Software v6.1.3.

### Comet assay (DNA damage) by single cell gel electrophoresis (SCGE)

For cellular DNA damage analysis, comet assay was performed on slides following the protocol developed by Singh et al [24] with slight modification. Briefly, 2.5 × 10^5^cells were seeded per well in 6-well plates. After 24 h of incubation, cells were detached by trypsinization and 2.5 × 10^4^cells were used for SCGE. Cells were mixed with 0.5% low melting agarose (LMA) and layered over pre-coated (1% NMA) glass slides. A third layer of LMA was then added to the slide. Cells were lysed by overnight incubation in lysis buffer at 4°C. Afterwards, slides were kept in an alkaline electrophoresis buffer for 20 min and then electrophoresed at 24 V with 300 mA current for 30 mins on ice. Next, slides were neutralised using neutralising buffer and stained with ethidium bromide. The slides were observed under fluorescence microscope. The degree of DNA damage was assessed by olive tail moment (product of tail length and the fraction of total DNA in tail). Comets were selected randomly and analysed using CASP software [25].

### Whole cell extraction and lysis for western blotting analysis

SH-SY5Y cells were seeded in a density of 5 × 105 cells in 6-well tissue culture plates for 24 h, pre-treated with 10 μM Mel for 1 h, followed by 50 μM Oxa for 24 h. Thereafter, the adherent cells were detached from the culture plate with gentle scraping using a cell scraper. The cells, including spontaneous floating and scraped cells, were centrifuged at 1,000 *g* and the cell pellet obtained was washed twice with PBS, followed by resuspension in 200 μl of ice cold lysis buffer [150 mM Sodium chloride, 10 mM Tris-HCl (pH 7.5), 0.5% Triton X-100, 0.1% SDS, 1 μl/ml of Protease inhibitor and 1X Phosphatase inhibitor (Sigma, USA)]. Cell lysates were sonicated for 2 min (75% energy, 20 secs on and 20 secs off) and centrifuged at 10,000 *g* for 20 min at 4 0C. The protein content was estimated by Bradford assay [26] with BSA as a standard using a micro plate reader (BioTek, USA) with Gen5 software.

### Western blotting analysis for apoptotic markers

Equal amounts of protein (30 μg) from each sample were boiled for 5 min in loading buffer [5% mercaptoethanol, 0.05% bromophenol blue. 75 mM Tris HCl (pH 6.8), 2% SDS and 10% glycerol]. Proteins were then run on 10 and 12% SDS polyacrylamide gels (BioRad, CA, USA) and the resolved proteins were electro blotted onto 0.45 μm PVDF membrane (Millipore, MA, USA) in a glycine/methanol transfer buffer (20 mM Tris Base, 150 mM glycine and 20% methanol) using the Trans Blot SD semidry transfer cell system (BioRad, CA, USA). The protein blots were blocked with 5% non-fat free dry milk in Phosphate-buffered saline (PBST) buffer containing 25 mM Tris—HCl, pH 7.4, 137 mM NaCl, 5 mM KCl, and 0.1% Tween 20 for 1 h at room temperature. Blots were probed with primary antibodies, rabbit anti-caspase 3 antibody (1:1000, Cell Signaling Technology, MA, USA), rabbit anti-cleaved caspase 3 antibody (1:1000, Cell Signaling Technology, MA, USA), rabbit anti-Poly (ADP Ribose) polymerase antibody (PARP, 1:1000, Cell Signaling Technology, MA, USA), rabbit anti-cleaved PARP antibody (Cell Signaling Technology, MA, USA), rabbit anti-Bax antibody (1:500, Santa Cruz Biotechnology, Inc., USA), mouse anti-Bcl-2 antibody (1:500, Santa Cruz Biotechnology, Inc., USA), and rabbit anti-GAPDH antibody (1:10000, Cell Signaling Technology, MA, USA) in PBST overnight at 4 0C. The blots were washed and incubated with horseradish peroxidase- conjugated secondary antibodies (Invitrogen, USA) for 1 h at room temperature. The membrane was developed and band visualization was executed using enhanced super signal chemiluminiscent substrate (Pierce, USA). Anti-GAPDH antibody was employed to stabilize protein as loading control. Densitometric analysis was performed using Image J software (1.50 version, NIH, USA).

### Cell fractionation for Bax localization and Cytochrome c (Cyt c) release

For mitochondrial and cytoplasmic isolation, 1 × 10^7^ SH-SY5Y cells were seeded in T75 flasks for 24–48 h. The cells were then exposed to 10 μM Mel for 1 h prior to the treatment of Oxa at 50 μM for 24 h. After 24 h, SH-SY5Y cells were then scraped and washed once with PBS. The cellular fraction was centrifuged at 1000 *g* for 10 min at 4°C. The resulting pellet was then re-suspended in isolation buffer (20 mM HEPES pH 7.4, 220 mM mannitol, 70 mM sucrose, 1 mM EDTA and 0.5 mM PMSF) and homogenized, with 10–15 strokes using a drill-fitted pestle. The homogenate was centrifuged at 800 *g* for 10 min at 4°C. The supernatant was then collected and centrifuged at 12,000 *g* for 15 min at 4°C. The resultant supernatant was separated and considered as cytoplasmic fraction. The mitochondrial pellet was further resuspended in the same above buffer and centrifuged at 10,000 *g* for 10 min at 4°C to yield an enriched mitochondrial fraction. The protein concentrations were quantified and equal amounts of protein were run on 12% SDS-PAGE gels for Bax localization and Cyt c release. Western Blot procedure performed as described above and probed with primary antibodies [rabbit anti-Bax and rabbit anti-Cyt c antibody (1:1000, Cell Signaling Technology, MA, USA)]. Rabbit anti- COX IV antibody (1:500, Santa Cruz) and mouse anti-β-actin antibody (1:1000, Santa Cruz Biotechnology) were used to validate the mitochondrial and cytoplasmic fractions, respectively.

### Statistical analysis

All statistical analyses were performed using Graph Pad Prism 5 software, USA. Data were analyzed using one way analysis of variance (ANOVA) followed by Tukey’s test. Results were represented as Mean ± SD or Mean ± SEM. and significance was defined as *P* ˂ 0.05.

## Results

### Mel rescued SH-SY5Y cells from Oxa induced cell death

**Fig 1:**
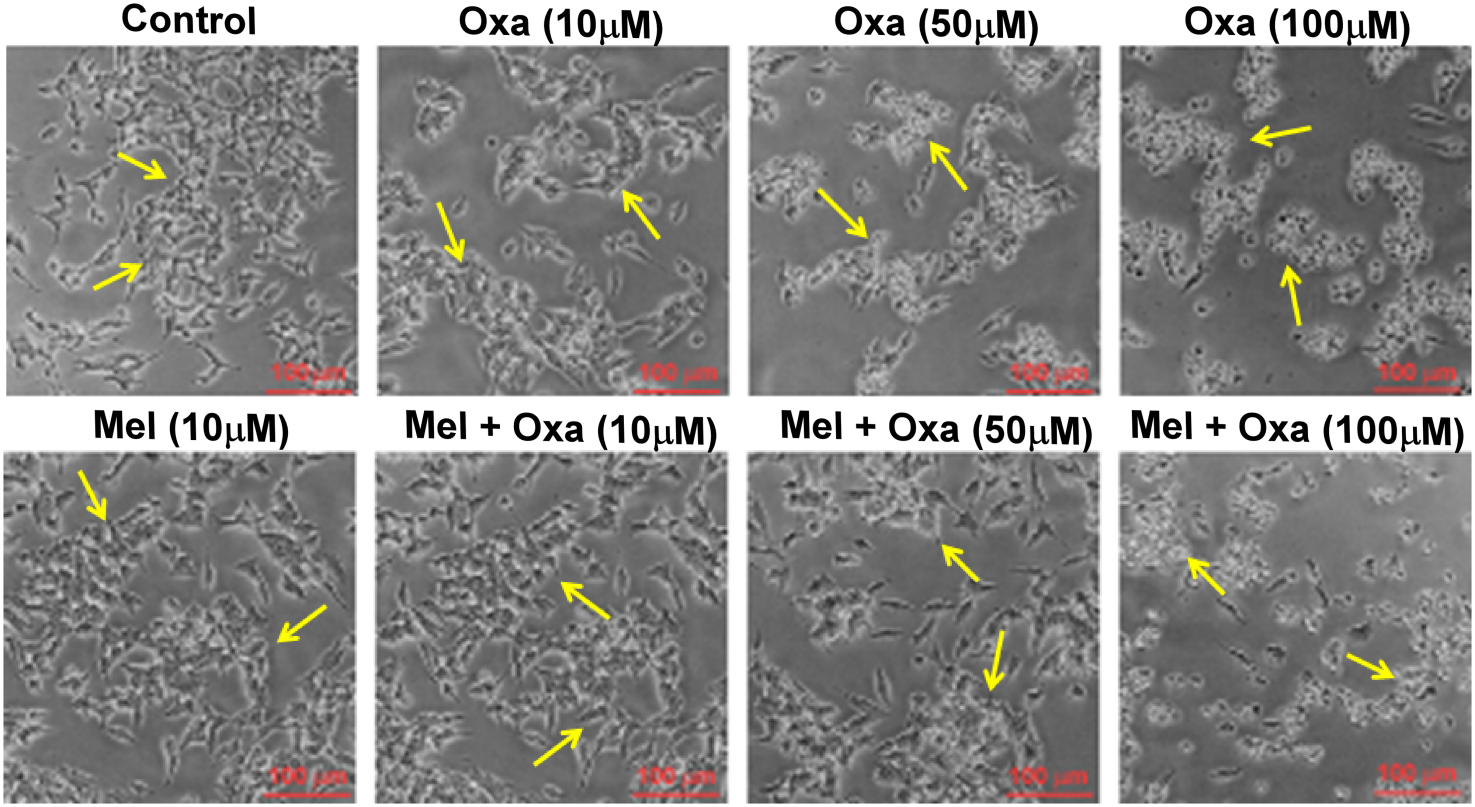
Protective effect of Mel on Oxa induced altered morphology and cellular damage. Comparative morphology of SH-SY5Y cells: Control, Mel, Mel + Oxa and Oxa treated groups. Bright field images of the cells were captured from at least six random fields. Yellow colored arrows depicts changes in morphology between groups.

**Fig 2:**
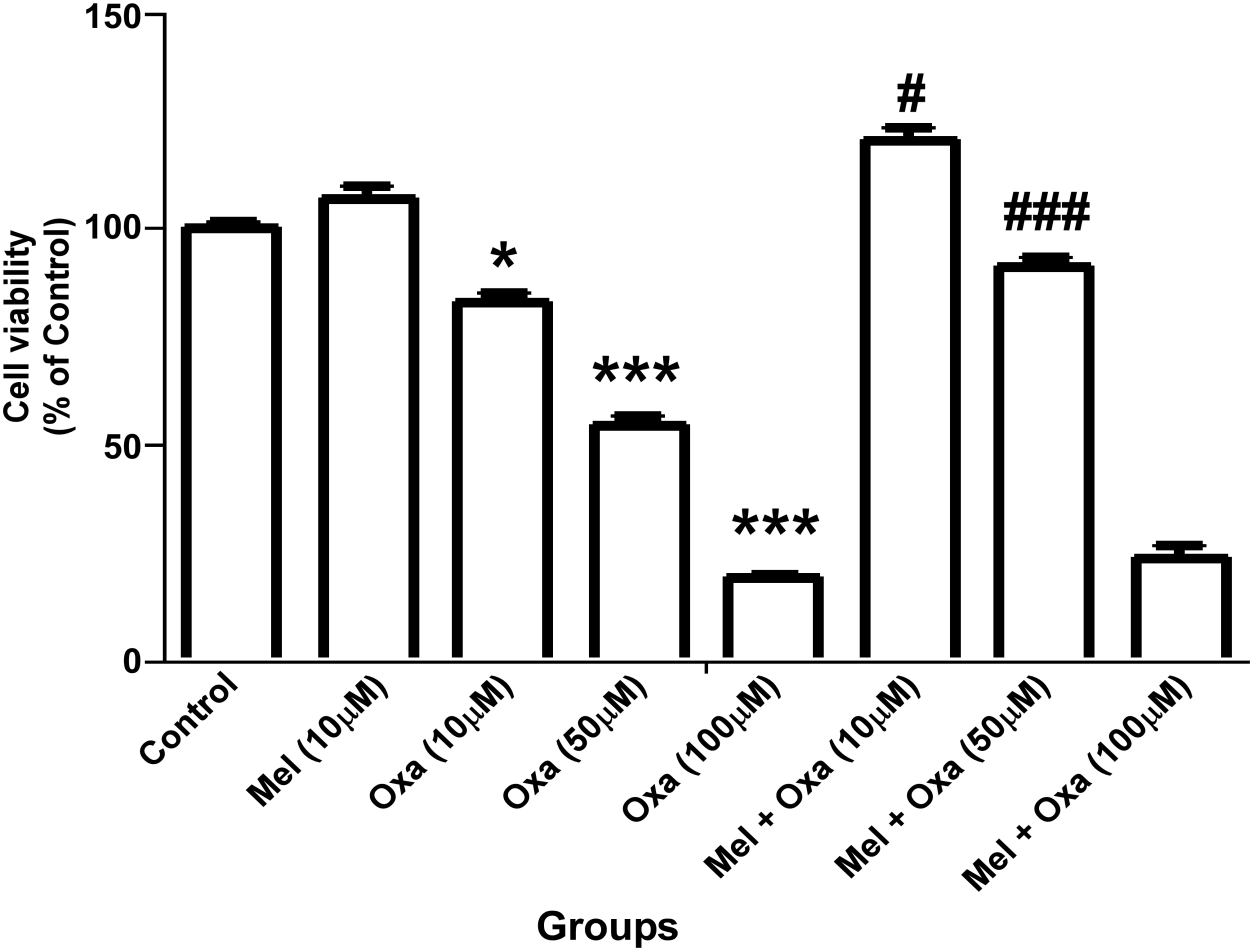
Protective effect of Mel on Oxa induced cytotoxicity assessed by mitochondrial reduction of MTT to formazan. Percentage of live cells were calculated and plotted as histogram. Data were represented as mean ± SE (n = 6) Significant difference (*P ˂ 0.05 and ***P ˂ 0.001) was indicated as compared to control and significant difference (#P ˂ 0.05 and ###P ˂ 0.001) was shown with compared to different concentration of Oxa treated groups. Cell viability was measured from at least three independent experiments.

SH-SY5Y cell viability was studied upon Oxa treatment at increasing doses (10 μM, 50 μM and 100 μM) using morphological analysis and MTT assay. Bright field images clearly demonstrated Oxa-exposed SH-SY5Y cells to display morphological alterations typically linked to an episode of apoptotic cell death. Changes observed in these images included cell shrinkage and membrane bleb formation, aggregation, and detachment from the cell surface. (Fig 1). Mel pre-treatment maintained cellular integrity and rescued the cells from Oxa induced altered cellular morphology. MTT cell viability assay further confirmed Oxa induced cellular damage. As shown in Fig 2, Oxa decreased SH-SY5Y cell viability significantly in a dose dependent manner (P ˂ 0.05—P ˂ 0.001) when compared to control. Mel pre-treatment significantly restored (P ˂ 0.05- P ˂ 0.001) cell viability in SH-SY5Y cells when compared to 10 μM and 50 μM Oxa treated cells. However, pre-treatment of 10 μM Mel was not shown to significantly replenish the cell viability when treated with 100 μM Oxa.

### Mel attenuated Oxa induced cytotoxicity in SH-SY5Y cells

**Fig 3:**
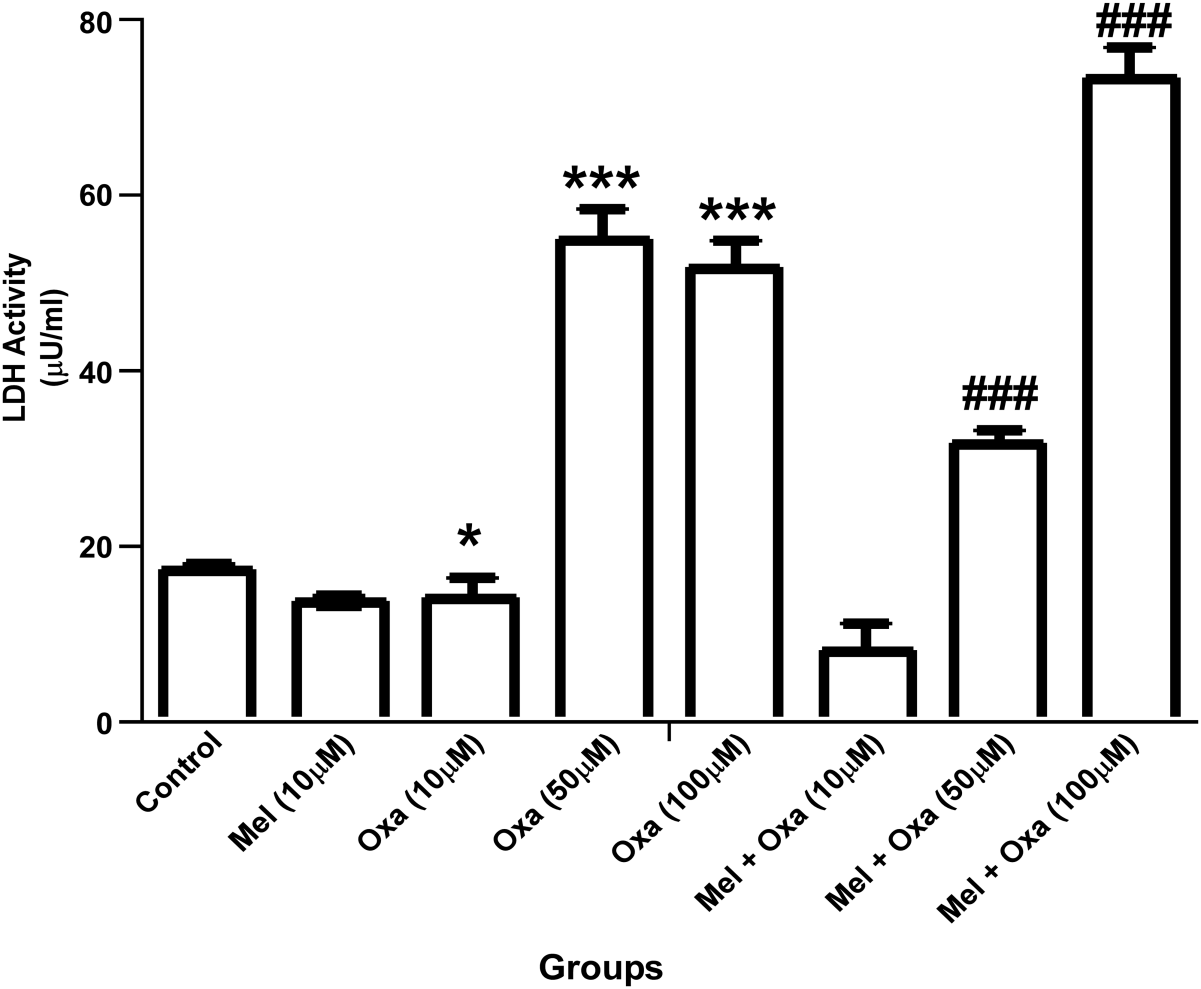
Protective effect of Mel on Oxa induced cytotoxicity in SH-SY5Y cells. Cytotoxicity was determined by LDH activity. Values were expressed as μU/ml of LDH activity. Data were represented as mean ± SE (n = 6) Significant difference (***P ˂ 0.001) was indicated as compared to control and significant difference (###P ˂ 0.001) was shown as compared to different concentration of Oxa treated groups. Cytotoxicity via LDH activity was measured with at least three independent experiments.

Since MTT assay is a quantitative measure of cell proliferation, a decrease in proliferating cells can result from either cell death or halted/slow proliferation. We utilized LDH cytotoxicity assay to quantitatively assess Oxa-induced cytotoxicity in SH-SY5Y cells. LDH activity was markedly raised in a dose dependent manner (P ˂ 0.05- P ˂ 0.001) in 10, 50, and 100 μM Oxa-treated SH-SY5Y cells when compared to control Group I (Fig 3). Mel pre-treatment significantly reduced (P ˂ 0.001) the LDH activity in SH-SY5Y cells as compared to 50 μM and 100 μM Oxa treated group. Of note, there were no significant differences observed in LDH activity in the Mel pre-treated group when compared to 10 μM Oxa treated cells. 50 μM Oxa treatment showed a nearly 50% decrease in cell viability. Therefore, 50 μM Oxa and 10 μM Mel were selected to be used in the subsequent experiments.

### Mel suppressed Oxa provoked cell death

**Fig 4:**
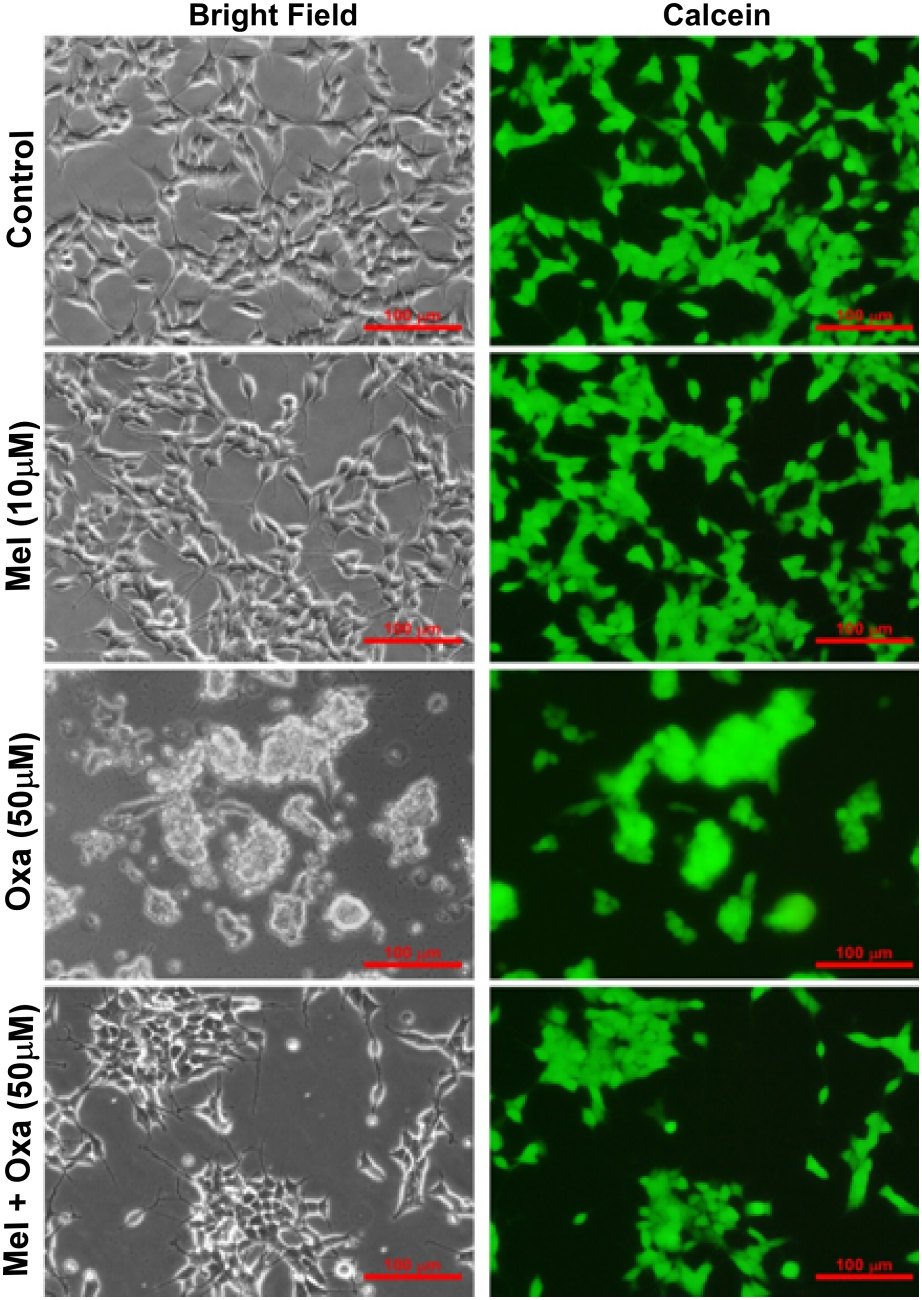
Effect of Mel pre-treatment on Oxa induced cytotoxicity by live cell assay in SH-SY5Y cells. Live cell assay was done by utilizing calcein fluorescent probe. Calcein AM can enter into cells and stains only live cells. Oxa treated cells showed shrinkage, round up and clumping indicative of loss of cell viability and progression towards death. Live Cell Assay was performed with at least three independent experiments.

Oxa induced cytotoxicity was additionally assessed utilizing Live Cell Assay. Calcein AM is converted by mitochondrial esterases into the green fluorescent probe calcein. We stained the cells with calcein to examine whether Oxa induced any sort of mitochondrial impairment. We treated the cells with 50 μM Oxa following 10 μM Mel pre-treatment for 1 h. Cells were then stained with Calcein for Live cell assay. Both bright field and fluorescent images were captured from same field. As seen before (Fig 1), bright field images clearly demonstrated altered cell morphology in Oxa treated SH-SY5Y cells when compared to control. However, Mel pre-treatment was shown to attenuate these alterations. Although calcein staining confirmed distorted morphology of Oxa treated cells and indicated cell death, no significant differences were observed in the fluorescence intensity across the groups (Fig 4), One possible theory to explain these findings considers the presence of apoptotic bodies or cell disintegration products that remained membrane bound following Oxa treatment. As calcein AM typically detects esterases within a membrane bound space, the presence of these apoptosis- byproducts could potentially elicit these insignificant fluorescence findings.

### Mel attenuated Oxa induced mitochondrial dysfunction

**Fig 5:**
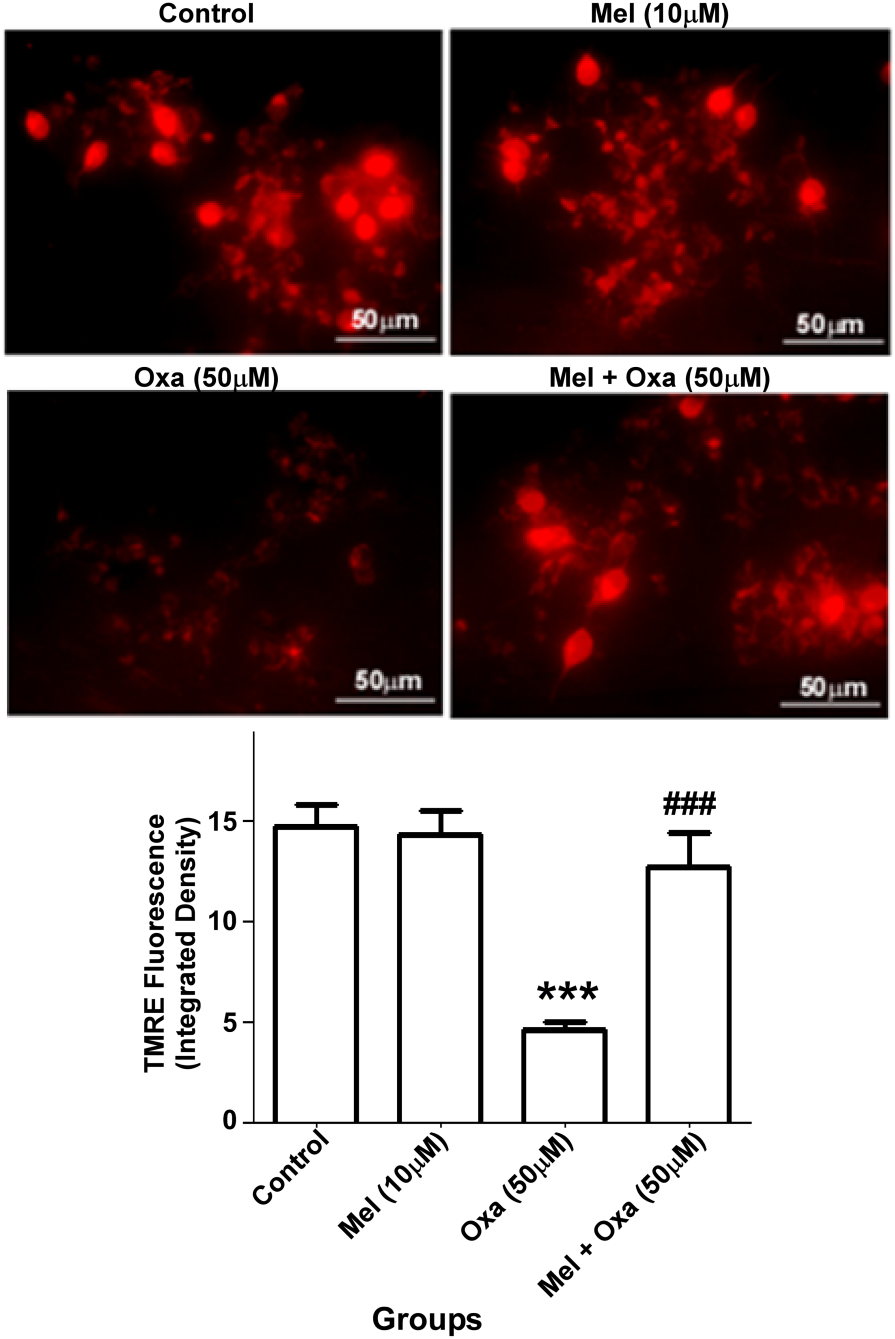
Effect of Mel pre-treatment on Oxa induced mitochondrial membrane depolarization in SH-SY5Y cells. Membrane depolarization was monitored by TMRE florescence integrated density. Data were represented as mean ± SE (n = 6) Significant difference (***P ˂ 0.001) was indicated as compared to control and significant difference (###P ˂ 0.001) was shown as compared to Oxa treated groups. Maximum projections were generated from focus planes and images were displayed with equal pixel intensity. The TMRE fluorescence integrated density was analyzed with Image J software (1.50 version, NIH, USA). Membrane potential was measured with at least three independent experiments.

To investigate if Oxa-induced cytotoxicity is mediated through mitochondrial dysfunction in our model system, we used TMRE, a fluorescent dye and indicator of mitochondrial membrane potential to check the mitochondrial membrane integrity and function. Oxa treated SH-SY5Y cells showed a significant decrease (P ˂ 0.001) in red florescence intensity compared to the control group. Cells pre-treated with 10 μM Mel displayed increased (P ˂ 0.001) red fluorescence intensity compared to the cells treated with Oxa alone (Fig 5). No significant differences were observed in the group of cells treated with Mel alone when compared with control Group I.

### Mel regulated Oxa activated ROS generation in SH-SY5Y cells

**Fig 6:**
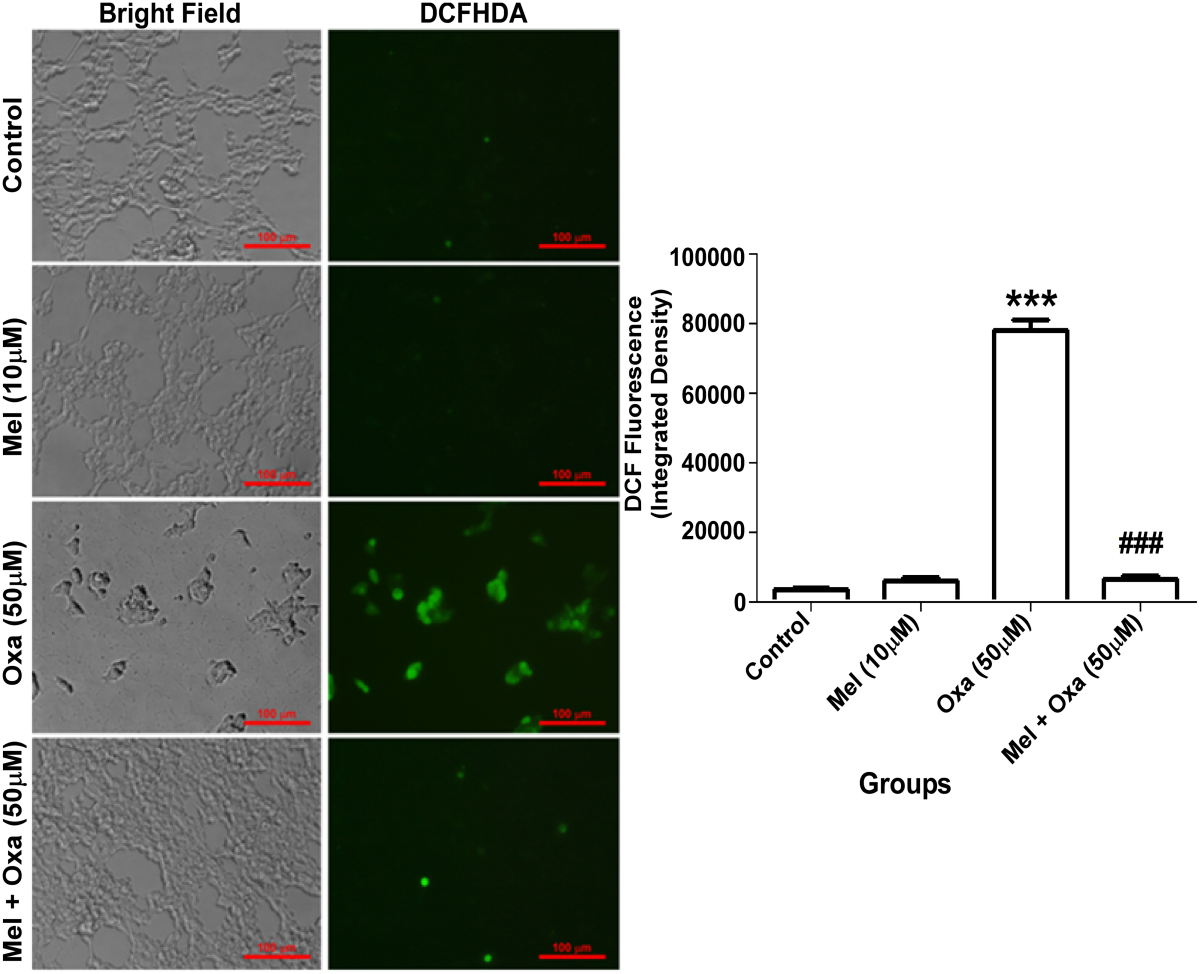
Inhibitory effect of Mel on Oxa induced intracellular accumulation of ROS and oxidative stress. ROS level were monitored by DCFHDA florescent dye. Data were represented as mean ± SE (n = 6) Significant difference (***P ˂ 0.001) was indicated as compared to control and significant difference (###P ˂ 0.001) was shown as compared to Oxa treated groups. ROS measurement was monitored by DCF florescence integrated density. Maximum projections were generated from focus planes and images were displayed with equal pixel intensity. The DCF florescence integrated density was analyzed with Image J software (1.50 version, NIH, USA). ROS accumulation was measured with at least three independent experiments.

To investigate if Mel modulates apoptosis or cell death by inhibiting ROS production, we measured the intensity of ROS generation using DCFDA as a fluorescent labelled probe. Oxa treatment significantly enhanced green fluorescence intensity (P ˂ 0.001) when compared to the control group (Fig 6). Pre-treatment of Mel showed complete restoration of ROS level as compared to 50 μM Oxa treated SH-SY5Y cells. In the presence of Mel treatment alone, the DCF florescence integrated density did not show any significant change in SH-SY5Y cells.

**Fig 7:**
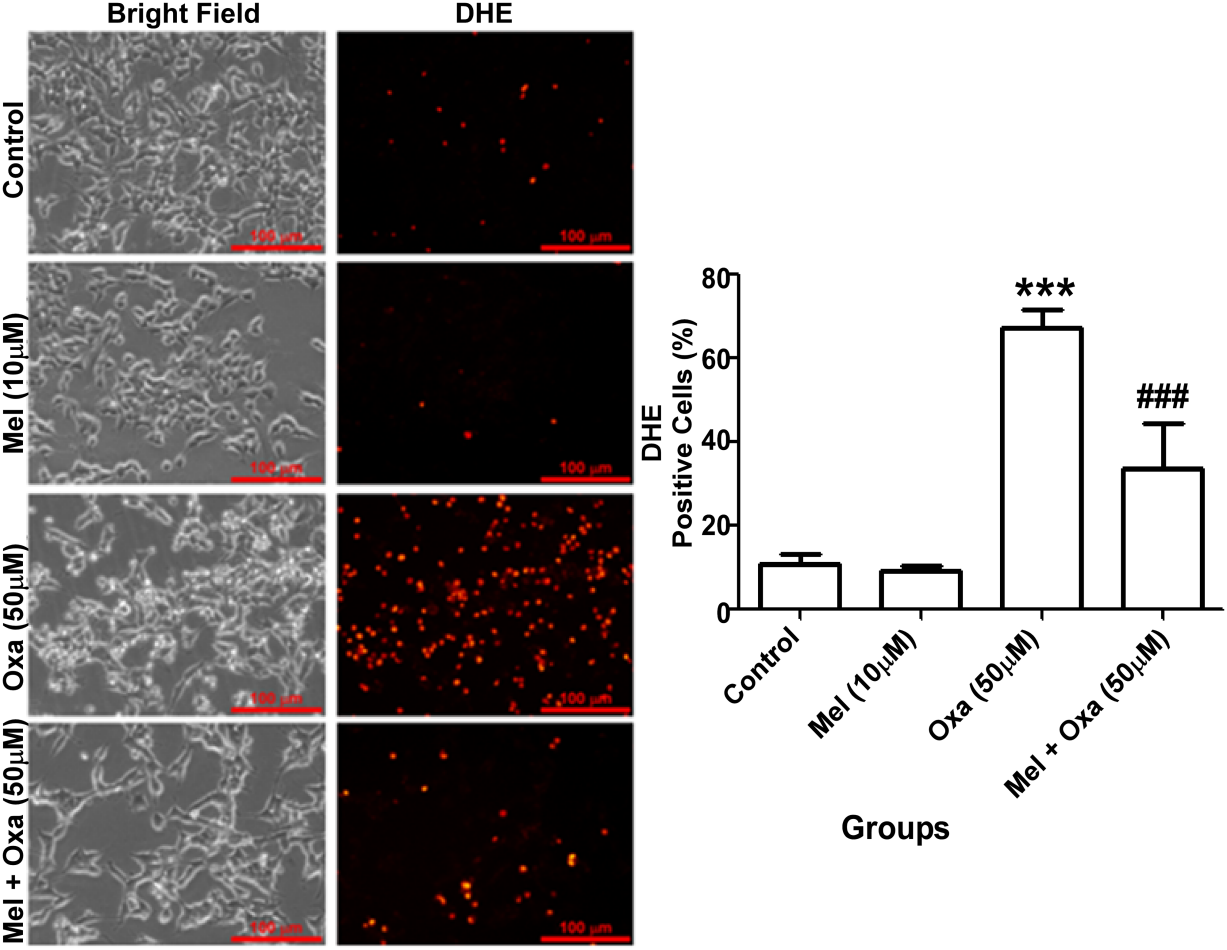
Protective effect of Mel on Oxa induced intracellular accumulation of superoxide anion and oxidative stress. Superoxide anion production was monitored by DHE florescent dye. Data were represented as mean ± SE (n = 6) and values were expressed as DHE stained positive cells. Significant difference (***P ˂ 0.001) was indicated as compared to control and significant difference (###P ˂ 0.001) was shown as compared to Oxa treated groups. The DCF fluorescence integrated density was analyzed with Image J software (1.50 version, NIH, USA). Five to six random images were captured at 20x magnification. Maximum projections were generated from focus planes and images were displayed with equal pixel intensity. Superoxide ion accumulation was quantified with at least three independent experiments.

To further validate Oxa induced ROS generation and protection conferred by Mel pre-treatment, we investigated the levels of superoxide anion generation by employing DHE florescent labelled probe. The lowest superoxide level was screened from the control group. Oxa treatment showed a majority of cell population (~ 70%, P ˂ 0.001) positive for DHE in comparison to the control group (Fig 7). Pre-treatment of Mel showed a significant reversal (~ 35%, P ˂ 0.001) of superoxide level when compared to 50 μM Oxa treated SH-SY5Y cells. Mel, by itself did not induce superoxide generation compared to control in SH-SY5Y cells.

### Involvement of Bax and Bcl-2 in Mel provoking mPTP opening induced by Oxa

**Fig 8:**
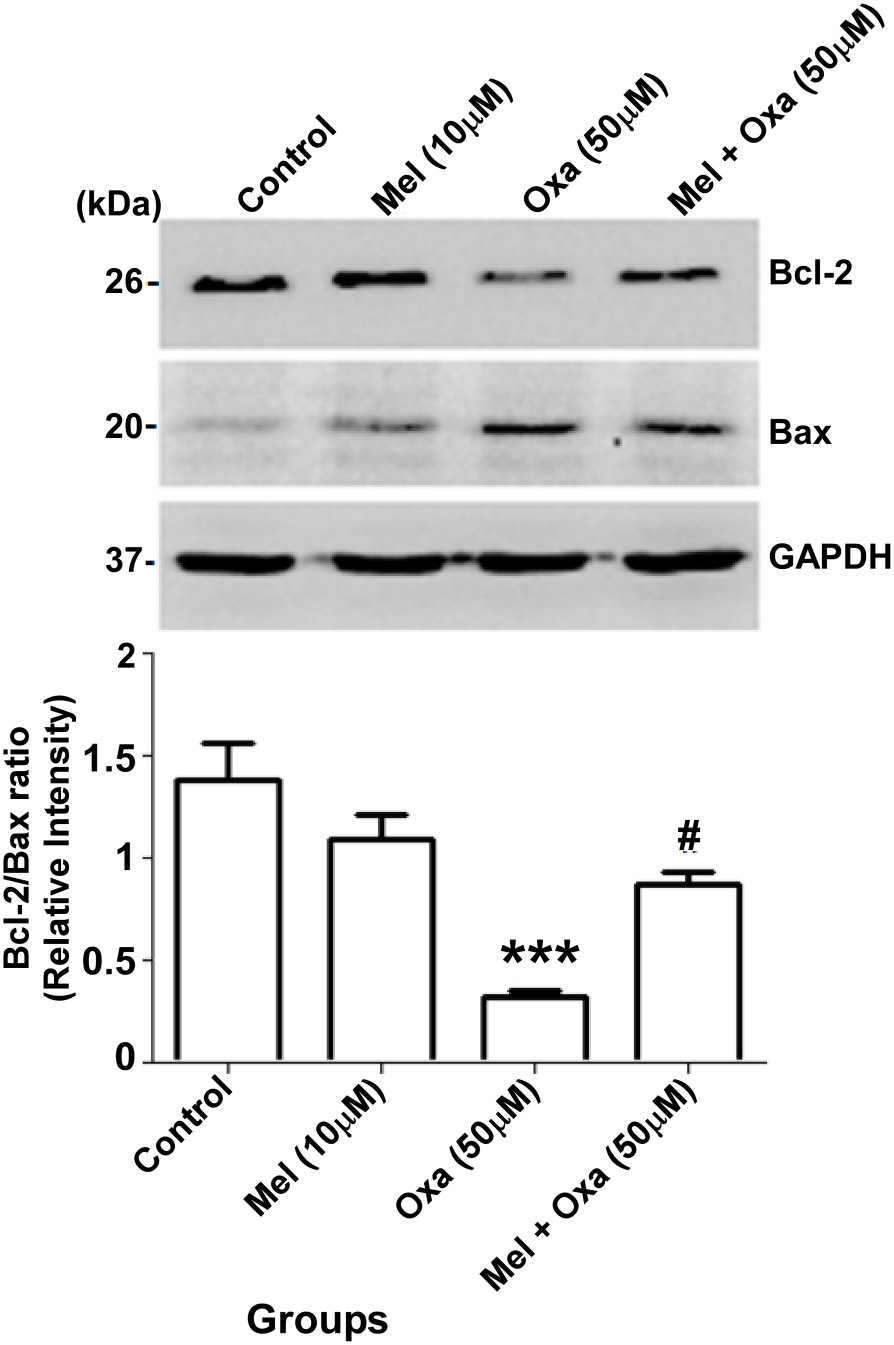
Protective effect of Mel on Oxa induced pro-apoptotic (Bax) and anti-apoptotic (Bcl-2) signal in SH-SY5Y cells. The quantification of immunoreactive bands for Bax and Bcl-2 realative to GAPDH and Bcl-2/Bax ratio were determined. Data were represented as mean ± SE (n = 6) and relative expression levels were quantified from four independent experiments. Significant difference (***P ˂ 0.001) was indicated as compared to control and significant difference (###P ˂ 0.001) was shown as compared to oxa treated groups. GAPDH level were monitored to verify equal amount of protein loading.

To investigate if Oxa induced cytotoxicity is mediated by apoptosis, we checked for the levels of Bcl-2 family of proteins. For this investigation, altered expression of Bax and Bcl-2 proteins were explored by Western blot analysis. Treatment with Oxa showed suppression of pro-survival protein Bcl-2 and simultaneous upregulation of pro-apoptotic protein Bax in SH-SY5Y cells (Fig 8). Bcl-2/Bax ratio, which determines the susceptibility of cells to apoptotic cell death, was markedly reduced (P ˂ 0.001) in Oxa treated group when compared to the control group. Cells pre-treated with Mel at 10 μM significantly antagonized Oxa-induced decrease in Bcl-2/Bax ratio (P ˂ 0.05).

### Bax translocation from cytosolic to mitochondrial fraction

**Fig 9:**
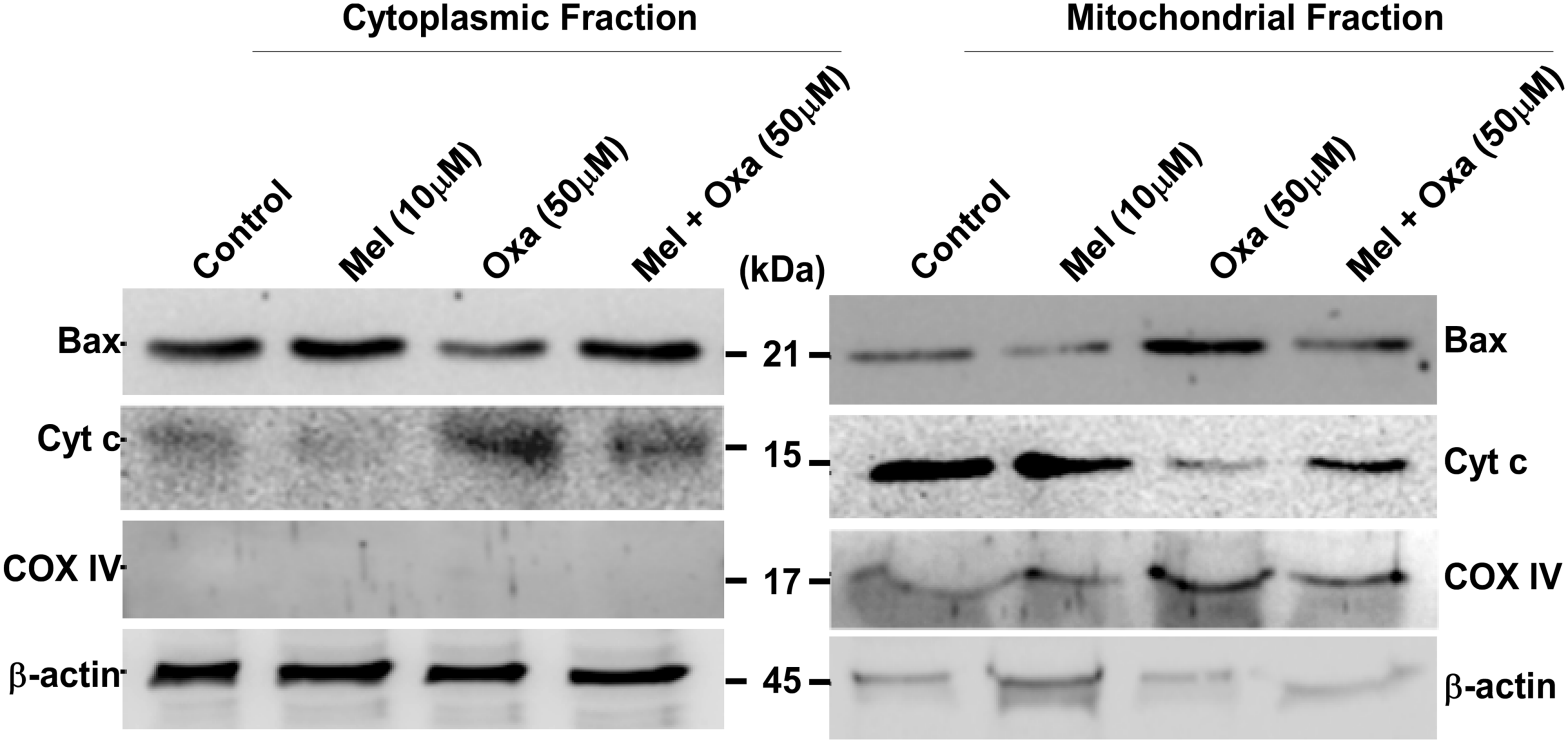
Representative immunoblots for Bax and Cyt c localization. Protective effect of Mel on Oxa induced Cyt c release into cytosol and Bax translocation from cytosol to mitochondria were evaluated in SH-SY5Y cell. Cells were pre-treated with 10 μM Mel for 1 h followed by with and without 50 μM Oxa for 24 h. COX IV and β-actin levels were monitored to verify equal amount of protein loading.

Apoptosis initiation is associated with translocation of inactive form of Bax from the cytoplasm to the mitochondria [27]. To determine if Oxa provoked Bax translocation to the mitochondria along with increased expression, we challenged the cells with 50 μM oxa and pre-treatment with 10 μM melatonin in their respective medium. In healthy control cells, Bax was primarly sequestered in the cytoplasm (Fig 9). Treatment with Oxa enhanced the loss of Bax levels in cytoplasmic fraction and concurrent rise in mitochondrial fraction. Alternately, 10 μM Mel pre-treatment rescued the cells from Oxa induced Bax translocation to mitochondria as compared to Oxa treated cells.

### Mel reduced Oxa induced Cyt c release from mitochondria in SH-SY5Y cells

Generally sequestered in the mitochondria, Cyt c release into the cytosol is regarded as a crucial initial phase in the mitochondria-mediated apoptotic cascade, occurring in the aftermath of bax translocation to the mitochondria. To further ascertain the role of Cyt c release in Oxa- treated cell apoptosis, we examined levels of Cyt c in the cytosol and mitochondria by Western blot. As shown in Fig 9, 50 μM Oxa did provoke release of Cyt c from mitochondria to cytosol in SH-SY5Y cells. Alternately, cells pre-treated with Mel retained Cyt c in the mitochondria, and lower levels were found in the cytoplasm as compared to Oxa-only treated SH-SY5Y cells.

### Mel inhibited Oxa induced apoptosis in SH-SY5Y cells

**Fig 10:**
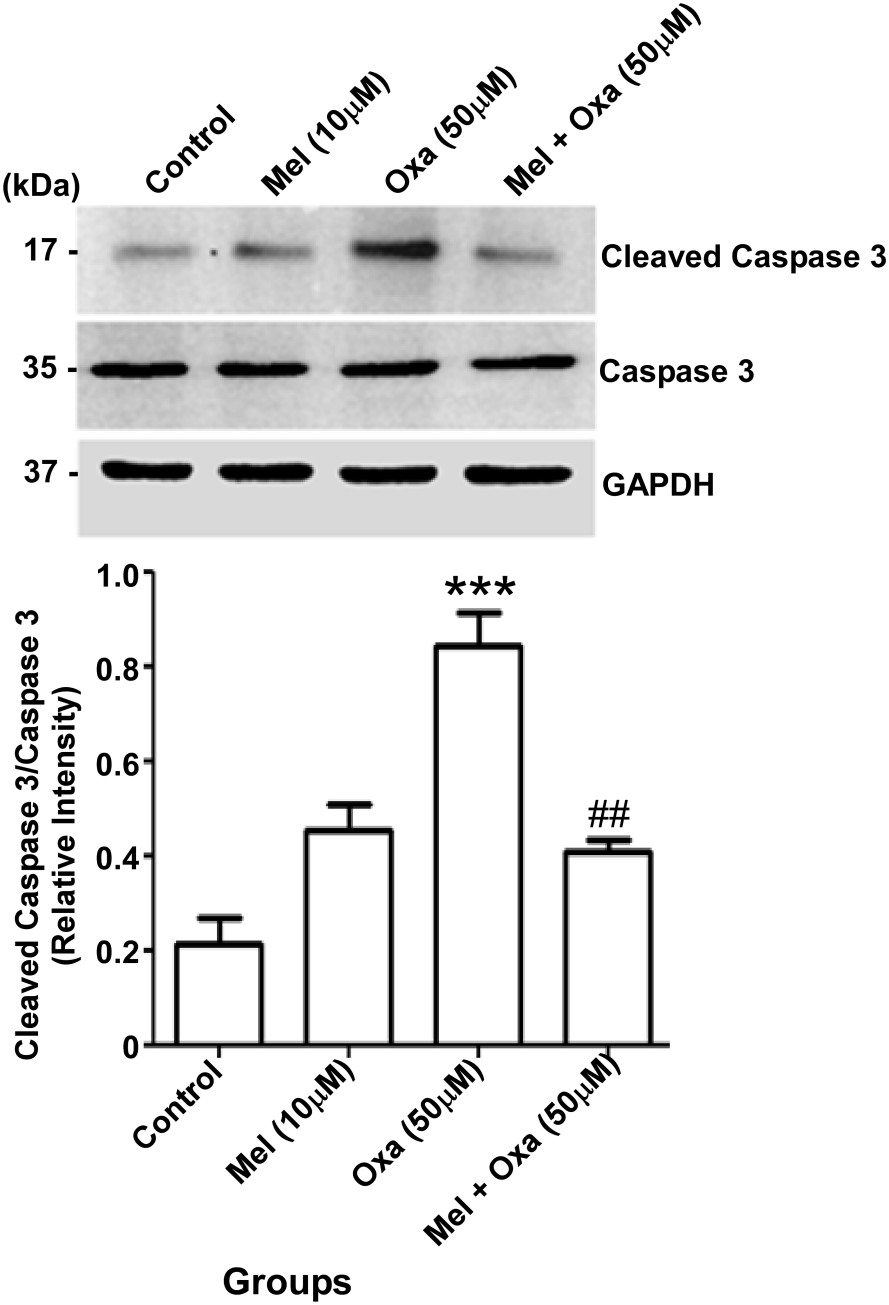
Protective effect of Mel on Oxa induced caspase 3 activation in SH-SY5Y cells. Data were represented as mean ± SE and relative expression levels were quantified from four independent experiments. Significant difference (***P ˂ 0.001) was indicated when compared to the control and significant difference (##P ˂ 0.01) was shown as compared to the Oxa treated groups. The quantification of immunoreactive bands for caspase 3 and cleaved caspase 3 relative to GAPDH and cleaved caspase 3/total caspase ratio were determined. GAPDH level were monitored to verify equal amount of protein loading.

Upon release to the cytoplasm, Cyt c activates Caspase 9, which in turn activates downstream effector Caspases 3 and 7 leading to apoptotic cell death [28]. To confirm Oxa induced apoptosis and anti-apoptotic effect conferred by Mel in Oxa-treated SH-SY5Y cells, we measured the expression of Caspase 3 and its activated cleaved product. As depicted in Fig 10, the ratio of cleaved Caspase 3: total Caspase 3 in Oxa treated group was markedly increased (P ˂ 0.001) in comparison to the control group. Pre-treatment of Mel at 10 μM showed significant (P ˂ 0.01) restored ratio of cleaved Caspase 3/total Caspase 3 when compared with the Oxa treated group.

### Mel counteracted Oxa induced PARP inactivation in SH-SY5Y cells

**Fig 11:**
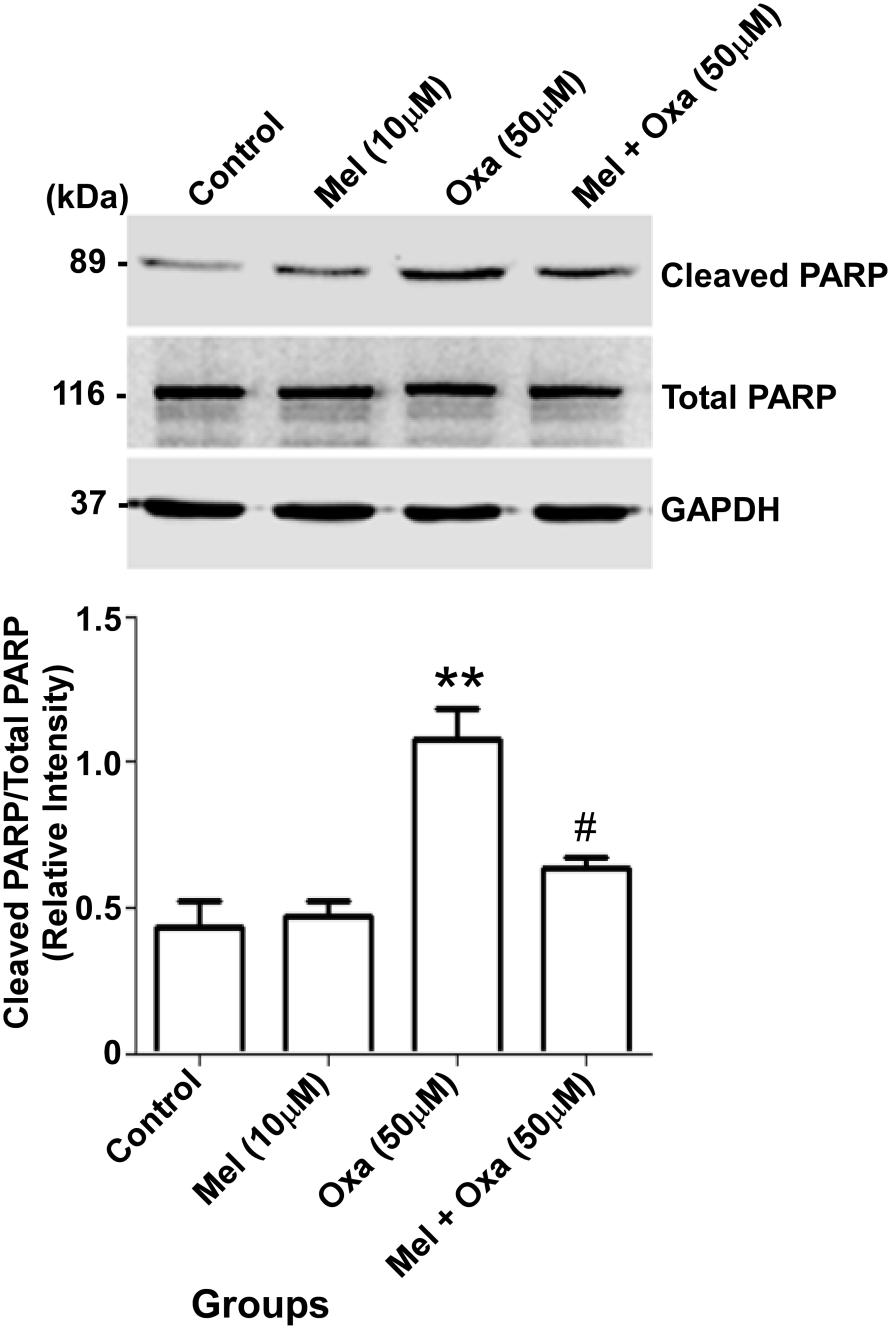
Protective effect of Mel on Oxa induced PARP inactivation in SH-SY5Y cell. Data were represented as mean ± SE and relative expression levels were quantified from four independent experiments. Significant difference (**P ˂ 0.01) was indicated when compared to control and significant difference (#P ˂ 0.05) was shown in comparison to the Oxa treated groups. The quantification of immunoreactive bands for PARP and cleaved PARP relative to GAPDH and cleaved PARP/total PARP ratio were determined. GAPDH level were monitored to verify equal amount of protein loading.

PARP is a nuclear target of cleaved caspase-3. Further, inactivation of PARP by proteolysis is considered to be a hallmark feature of apoptosis [19]. Since increased activating proteolysis of caspase 3 was noticed in Oxa treated SH-SY5Y cells, we evaluated PARP proteolysis by Western blot analysis. As shown in Fig 11, Oxa treatment induced significant PARP cleavage with accumulation of 89 kDa inactive cleaved product leading to elevated ratio of cleaved PARP/total PARP (P ˂ 0.01) in comparison to the control group. Pre-treatment of Mel at 10 μM significantly (P ˂ 0.05) attenuated Oxa induced PARP cleavage when compared with the Oxa treated group.

### Mel protected SH-SY5Y cells from Oxa induced apoptosis

**Fig 12:**
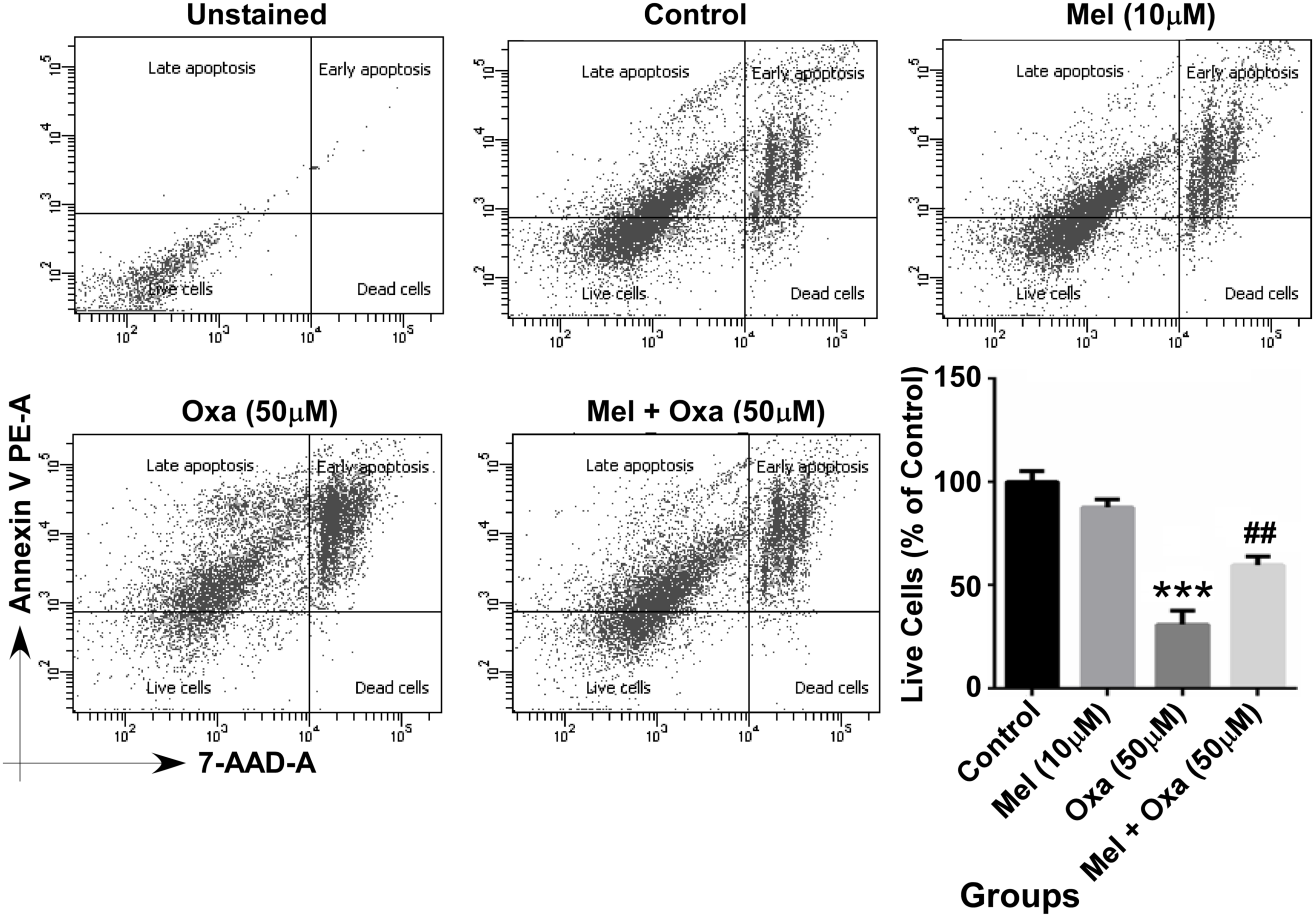
Protective effect of Mel on Oxa-induced apoptosis by flow cytometric analysis. Data were represented as mean ± SE from four independent experiments. The number in the lower right quadrant signifies the percentage of early apoptotic cells, and upper right quadrant signifies the percentage of late apoptotic cells. Significant difference (***P ˂ 0.001) was indicated as compared to control and significant difference (##P ˂ 0.01) was shown when compared to the Oxa treated groups.

Protection conferred by Mel pre-treatment against Oxa induced apoptotic cell death was further evaluated by flow cytometric analysis. We performed PE-Annexin V staining in conjunction with 7-Amino-Actinomycin (7-AAD). Cells were treated with 50 μM Oxa with and without 10 μM Mel pre-treatment and were stained with PE-Annexin V and 7-AAD to assess cell death. PE-Annexin V binds to cells in early stages of apoptosis and the fluorescent dye 7-AAD can only pass through membranes of dead and damaged cells. Therefore, it is useful in identifying cells undergoing late stages of apoptosis or that are already dead. Cells stained negative for both PE-Annexin V and 7-AAD were considered viable (left lower R3 quadrant). As displayed in Fig 12, exposure to Oxa resulted in a significant decrease in live cells percentage (~ 30%, P ˂ 0.001) compared to the control group. In contrast, Mel pre-treatment significantly reversed the percentage of live cell (~ 60%, P ˂ 0.001) when compared to Oxa treated cells.

### Mel attenuated Oxa induced DNA single strand breaks in SH-SY5Y cells

**Fig 13:**
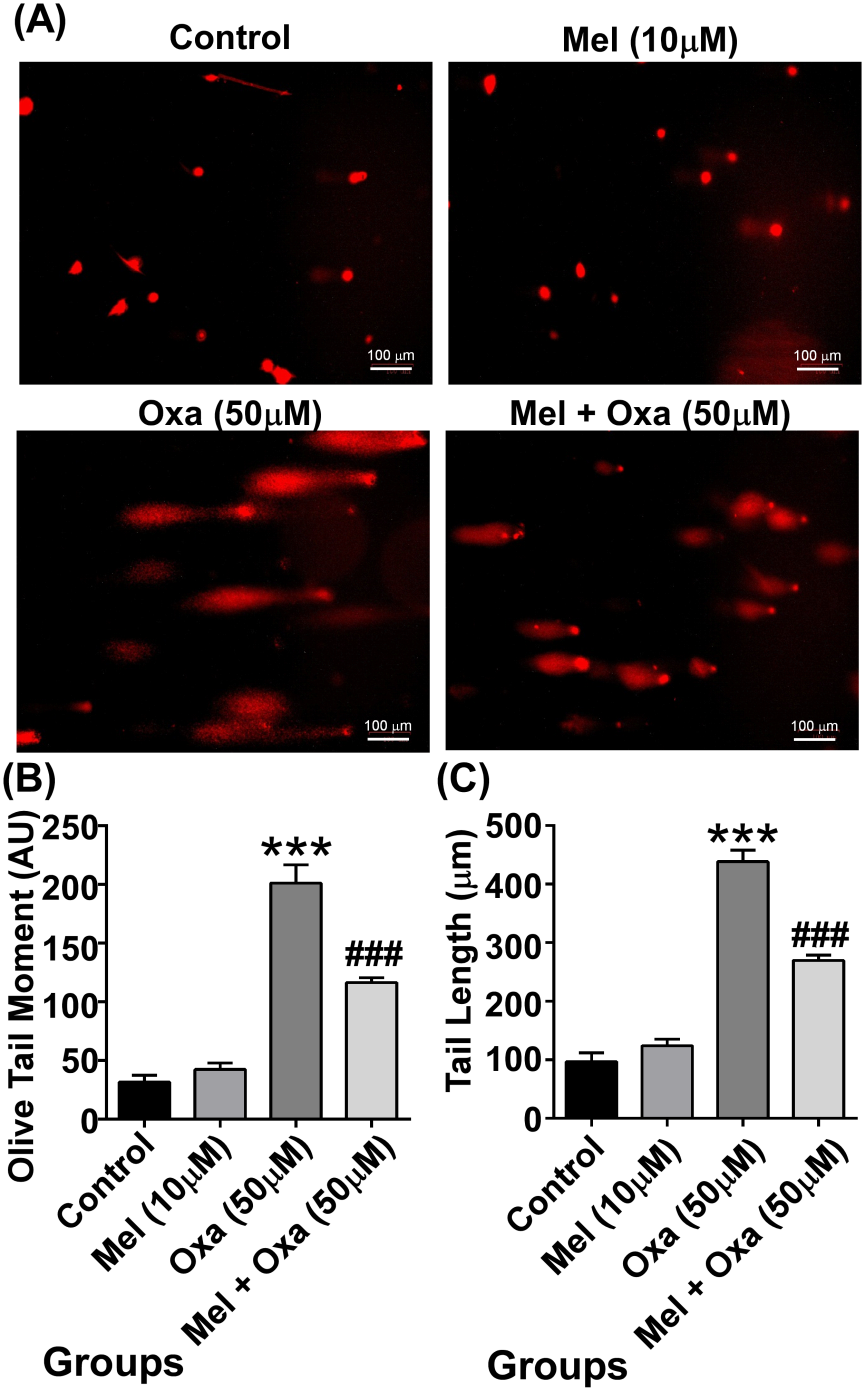
Protective effect of Mel on DNA damage induced by Oxa. Representative images (A) of comet assay on SH-SY5Y cells after exposure to Oxa (50 μM). Oxa incurred DNA damage leading to more number of comet formation compared to Mel pre-treatment cells. DNA damage in cells was quantified as olive tail moment (B) and tail length (C). Significant difference (***P ˂ 0.001) was indicated in comparison to control and significant difference (##P ˂ 0.01) was shown when compared to Oxa treated groups.

DNA is vulnerable to damage resulting from various insults including genotoxic cancer therapeutics; further, failure in DNA repair mechanisms can cause cell death. Results so far confirmed Oxa-induced apoptotic cell death in SH-SY5Y cells could be mitigated with Mel pre-treatment. To ascertain if Oxa-induced apoptosis involved DNA damage and, in addition, whether Mel pre-treatment protects against DNA damage, we utilized the comet assay, a standard method to assess DNA damage. DNA damage was recorded in terms of tail length and olive tail moment in the cells from all treatment groups. As such, the longer the tail length measured, the greater the degree of DNA damage. Olive tail moment, a measure of the amount of DNA in the tail and distribution of DNA in the tail, is a common descriptor along with tail length. Fig 13 shows signs of DNA damage in Oxa exposed cells when compared to control and Mel pre-treated cells. Oxa treated cells showed significantly increased (P ˂ 0.001) olive tail moment as well as comet tail length when compared to control cells. Mel pre-treatment (P ˂ 0.001) inhibited Oxa-induced DNA damage, showing decreased olive tail moment and shorter tail length in contrast to Oxa treated cells.

## Discussion

The anti-cancer properties of platinum compounds, as well as the neurotoxic effects of this agents, are contingent upon their capacity to form adducts with DNA [1, 2, 29] and cytoplasmic proteins [3, 4]. Results from the present study clearly suggested that Oxaliplatin (Oxa), a platinum-based anti-neoplastic agent, exerts cytotoxic effects to SH-SY5Y human neuroblastoma cells in a dose-dependent manner. Currently, multiple reports on the neurotoxic properties of Oxa exist; however, there is less reported evidence regarding the specific roles of mitochondrial pathways involved in the apoptotic cascade and neurotoxicity induced by this drug. The results of this study exposed plausible mechanisms of cytotoxic damage induced by Oxa treatment to SH-SY5Y cells. In addition, our study proposed Melatonin (Mel), a hormone derived from the pineal gland, as a powerful neuroprotective agent owing to its anti-oxidant and free-radical scavenging properties. The findings in this study supported this evidence as pre-treatment of SH-SY5Y cells with Mel rescued the cells from Oxa-induced cytotoxicity. Platinum compounds induce apoptosis in post-mitotic DRG neuronal cultures by interfering with neurons ability to re-enter the cell cycle [5, 30]. AS SH-SY5Y human neuroblastoma cells are generally considered as immature neuroblasts, This property of platinum compounds make SH-SY5Y human neuroblastoma cells a reliable model for studying neurotoxic damage induced by Oxa, as SH-SY5Y human neuroblastoma cells are generally considered to be immature neuroblasts [31]. Moreover, these immature neuroblasts are useful in measuring cytotoxic damage despite the fact they are still in the dividing phase [6]

Our results from MTT and LDH cytotoxicity assays showed Oxa- induced cytotoxicity in a dose-dependent manner in SH-SY5Y cells ranging from 20% cell death at 10 μM to 50 μM and ~ 70% cell death at 50 and 100 μM Oxa, respectively, measured at 24 h post treatment. Mel was found to efficiently rescue SH-SY5Y cells from Oxa induced neurotoxic insult, and pre-treatment of cells with Mel at a concentration of 10 μM significantly inhibited Oxa-induced cytotoxicity. This protective effect was noted in SH-SY5Y cells that were treated with up to 50 μM of Oxa. Furthermore, Mel pre-treatment helped cells to maintain a healthy morphology, evidenced by the reduced appearance of apoptotic features (such as membrane blebbing, shrinkage of cells, and detachment of cell surfaces), following exposure to Oxa [32–34]. Findings from our study are in agreement with previously published studies which have also found Oxa treatment to reduce viability of SH- SY5Y cells [1, 35]. We found 50 μM Oxa reduced SH-SY5Y cell viability by nearly 50%. In addition, 10 μM Mel efficiently rescued the cells from Oxa-induced cytotoxicity. From these findings, we determined 50 μM Oxa and 10 μM Mel to be standard doses for the subsequent studies. Survival of the cells was further assessed by live cell assay using calcein AM, as it can easily pass cell membranes where it is then converted to green fluorescent calcein by cytoplasmic esterases. From this method, live cells with intact membranes could be identified by green fluorescence. Strikingly, despite morphological features indicative of apoptosis, Oxa treated SH-SY5Y cells also exhibited green fluorescence. One theory we propose to explain these findings accounts for the presence of apoptotic bodies and disintegrated cell fragments. As calcein AM detects esterases within closed membrane spaces, perhaps membrane enclosed apoptotic fragments also allowed for the conversion of green fluorescent calcein.

In the present study, Oxa treatment inhibited TMRE fluorescence intensity in SH-SY5Y cells indicative of decreased Δψm and mitochondrial membrane impairment. TMRE, a cationic lipophilic dye enters into the cell as an ester which is consequently hydrolyzed and the final product, tetramethylrhodamine, then accumulates in the mitochondria due to a high membrane potential [36]. Oxa induced mitochondrial membrane depolarization or impairment was markedly reversed by pre-treatment of Mel in SH-SY5Y cells, allowing for integrity of the Δψm to be maintained. This observation, similar to previously published reports, provides further evidence of the effect of Oxa in reducing membrane potential neuroblastoma SH-SY5Y cells [1, 37]. Next, we determined whether Oxa treatment provoked ROS production utilizing a green fluorescent probe DCFDA. Our results illustrated increased intracellular ROS production indicated by increased fluorescence in Oxa treated SH-SY5Y cells. Consequently, this ROS generation may result in DNA damage and oxidation of lipids and proteins in the cell. In line with its anti-oxidant property, pre-treatment with Mel significantly mitigated Oxa induced ROS production.

Previous reports have observed ROS overproduction to occur rapidly after neuronal dysfunction, an important pathway to recognize as these reactive species are involved in both necrotic and apoptotic cell death [38–40]. It is well known that mitochondria play a crucial role in sustaining the oxidation-reduction equilibrium [41, 42]. In our study, we found that superoxide or intracellular ROS in Oxa-treated SH-SY5Y cells to be markedly increased compared to the control cells. In addition, the overproduction of ROS amplified mitochondrial impairment by promoting Δψm collapse, rupture of mitochondrial membranes, and suppression of mitochondrial complex machinery [43, 44].

Mel has been shown to exhibit competent antioxidant activity. Evidence of this property has been shown by the ability of Mel to directly quench a variety of ROS species, including singlet oxygen, hydroxyl radical, peroxyl radicals and superoxide anion and H2O2 in SH-SY5Y cells [45, 46]. A possible event in early apoptosis stages is the appearance of phosphatidyl serine on the outer surface of the cell membrane. Annexin V primarily binds to phosphatidyl serine and is capable of identifying phosphatidyl serine on apoptotic cell platforms. PI has the capacity to diffuse into necrotic cells, and was employed in our study as an indicator of reduced cellular membrane integrity. Membrane permeability with both Annexin V and PI staining highlighted the effect of Oxa in significantly impairing SH-SY5Y cell membrane integrity. Alternately, Annexin V and PI staining identified the reverse of these membrane permeability findings in SH-SY5Y cells pre-treated with Mel.

Exposure of cells to apoptotic stimuli drives Cyt c release from mitochondria into the cytosol, thereby activating the cell death proteases. Thus, Cyt c release from the mitochondria into the cytosol can serve as an indicator for induction of apoptosis. Our results depicted that the activation and release of Cyt c into the cytosol was markedly enhanced in the Oxa targeted cells compared to control cells. Mel pre-treatment was found to significantly inhibit the mitochondrial to cytosol transfer when compared to Oxa-treatment alone. This observation is similar to previous studies that have also found Mel to downregulate Cyt c in SH-SY5Y cells challenged with neurotoxicants [45, 46].

Since Oxa was shown to induce mitochondrial membrane impairment in SH-SY5Y cells, and Bcl-2 family of proteins are key regulators of the mitochondrial outer membrane permeability [47], the regulation of apoptotic pathways were induced by multiple stimuli. We measured the expression levels of two major Bcl-2 family of proteins- anti-apoptotic member, Bcl-2 and pro-apoptotic member, Bax. In our model of SH-SY5Y cells, exposure to Oxa markedly reduced Bcl-2 protein levels and simultaneously enhanced Bax expression resulting in a marked decrease in Bcl-2/Bax ratio. Importantly, this ratio acts as a key regulator of the apoptotic switch; therefore, it makes sense for this aberrant Bcl-2: Bax expression to be seen in the setting of upregulated apoptosis. Reduced Bcl-2 level may be due to proteolytic cleavage by caspase3/7 or transcriptional downregulation. Caspase-dependent cleavage of Bcl-2 can be identified by a 23 kDa fragment [31]; although, this was not detectable in our immunoblots as the antibody used was not specific for this fragment. Interestingly, SH-SY5Y cells exposed to Oxa did demonstrate Caspase 3 activation by a prominent ~ 4 fold increase. During the pro-apoptotic state, Bax progresses from the cytosol to the outer mitochondrial membrane, where it is capable of binding with mitochondrial proteins to provoke the mPT pore opening. [35, 48]. In this same way, our study also observed a significant translocation of Bax from the cytosol to the mitochondria upon exposure to Oxa. Of note, Mel pre-treatment not only was effective in reversing the Oxa-mediated decrease in Bcl-2/Bax ratio, it also attenuated Bax translocation to mitochondria and Caspase 3 cleavage. These results strongly emphasize the efficacy of Mel as a neuroprotectant through its action in suppressing the upregulation of Bax, promoting Bcl-2 expression, and ultimately blocking mPT pore opening. Further, the restored ratio of Bcl-2/ Bax is further proof of the ability of Mel pre-treatment to block mPT pore opening [49]. One possible mechanism in Mel pre-treatment blockade of mPT pore opening in Oxa-exposed cells may involve other pathways modulated by this antioxidant. Perhaps, other pathways of Mel protection, such as decreased intracellular ROS generation and modifications in Bax and Bcl-2 protein expression, also play a role in this action.

**Fig 14:**
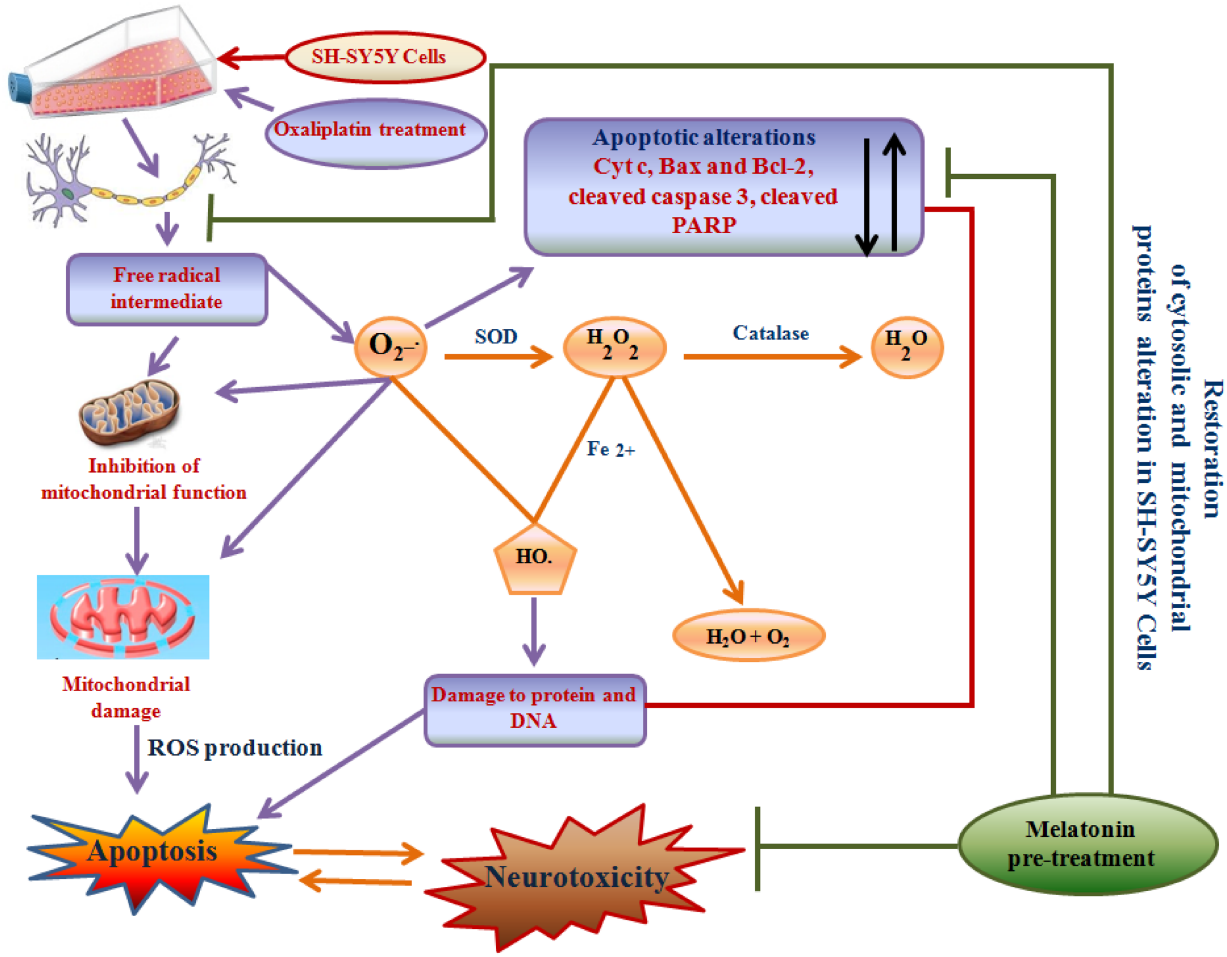
Role of oxidative stress in oxaliplatin induced neurotoxicity. Increased intracellular ROS may result in DNA damage, oxidation of lipids and proteins. This can elicit various alterations in neurons like redundant mitochondrial damage, activation of caspase 3 a inactivation of PARP leading to apoptosis. Melatonin alters this apoptotic signaling by restoring mitochondrial function, scavenging free radicals and triggering anti-apoptotic signaling.

Our results demonstrated the Oxa-induced neurotoxic effects in SH-SY5Y cells to involve mitochondrial membrane potential in conjunction with activation of caspase 3. As caspase 3 is a key substrate for Cyt c, we checked for cellular localization of Cyt c [50]. Oxa treatment increased the levels of Cyt c in the cytosol. However, Mel pre-treatment in SH-SY5Y cells was shown to attenuate this response. Simultaneously, our results also suggested a defense mechanism conferred by Mel pre-treatment in limiting the release of Cyt c and inhibiting apoptosis-related protein activation in Oxa treated SH-SY5Y cells [51]. It has been observed that oxaliplatin-induced ROS generation may elicit various alterations in neurons such as redundant mitochondrial damage, activation of caspase 3, inactivation of PARP, and apoptosis. PARP is a well-known nuclear target of cleaved caspase-3; further, proteolytic cleavage of PARP is considered to be a hallmark feature of apoptosis [19, 52, 53–56]. Since increased proteolytic activation of Caspase-3 was found in Oxa treated SH-SY5Y cells, we studied the proteolysis of PARP. In our results, the ratio of cleaved PARP/total PARP in Oxa treated group was significantly enhanced when compared to control. Pre-treatment with Mel significantly attenuated PARP cleavage, thereby restoring cleaved PARP/total PARP ratio in the cells. As apoptosis is concomitant of failure to repair DNA damage, we examined DNA damage incurred by Oxa in our model system. Our results demonstrated an increased DNA damage assessed by comet tail length and olive tail moment, which were significantly enhanced in Oxa exposed cells compared to the control cells. Mel pre-treatment markedly reduced the comet tail length and olive tail moment when compared to the Oxa treated group, protecting the cells from DNA damage. Reports have indicated that ROS may cause DNA base modifications and strand breaks. An estimated 20 base modifications, such as thymine glycol, 5-hydroxy methyl uracil, 8-hydroxyladenine, and 7-methyl-8-hydroxyguanine, have already been reported. Our results highlight the neuroprotective role of Mel by demonstrating its ability to mitigate Oxa-induced ROS production. In this way, Mel prevents DNA strand break, restores mitochondrial function, scavenges free radicals, and triggers anti-apoptotic signaling in SH-SY5Y cells (Fig 14). Proteolysis of PARP in concert with proteolytic activation of caspase 3 strongly suggests the involvement of apoptosis in Oxa-induced neurotoxic damage to SH-SY5Y cells. Importantly, the ability of Mel pre-treatment to rescue cells from Oxa-induced apoptosis establishes this pineal hormone as a potent neuroprotectant.

We could also show the protective effect of 10 μM melatonin on oxidative stress parameters associated with oxaliplatin treatment, as beneficial effects were seen on Tail length DNA in comet assay. ROS can introduce many DNA base modifications and strand breaks. In the current study, melatonin was proven to be a direct-acting antioxidant and anti-apoptotic molecule, clearly demonstrated through its ability to restore mitochondrial functions by scavenging free radicals and regulating apoptosis in SH-SY5Y cells.

## Conclusion

Melatonin can provide neuroprotection by both direct and indirect anti-oxidative activities. Further, it has an anti-apoptotic role in toxic insults caused by chemicals like oxaliplatin. Melatonin provides protection by modulating mitochondrial functions and apoptosis-regulatory proteins such as Cyt c, Bax, Bcl-2, caspase-3 activation, and PARP. Although further studies are required to decipher the mechanism of action of melatonin, these results suggest a protective role of melatonin as therapy against chemicals able to induce mitochondria-mediated toxicity.
